# Metabolic and Metabolomic Insights Regarding the Omega-3 PUFAs Intake in Type 1 Diabetes Mellitus

**DOI:** 10.3389/fmolb.2021.783065

**Published:** 2021-12-03

**Authors:** Carmen Purdel, Anca Ungurianu, Denisa Margina

**Affiliations:** ^1^ Department of Toxicology, Faculty of Pharmacy, Carol Davila University of Medicine and Pharmacy, Bucharest, Romania; ^2^ Department of Biochemistry, Faculty of Pharmacy, Carol Davila University of Medicine and Pharmacy, Bucharest, Romania

**Keywords:** omega-3 PUFAs, dietary intake, EPA, DHA, type 1 diabetes mellitus, metabolomics

## Abstract

Type 1 diabetes mellitus (T1DM) is currently considered an autoimmune disease characterized by the destruction of pancreatic β-cells, insulin deficiency, and dysglycemia. Dietary factors, including omega-3 polyunsaturated fatty acids (ω-3 PUFAs), were reported to influence T1DM. Therefore, a better understanding of the potential role of ω-3 PUFAs in the development and progression of T1DM will help to improve the clinical management of the disease. In this review, we explored the current understanding of molecular mechanisms and signaling pathways induced by ω-3 PUFAs and the beneficial effects of ω-3 PUFAs intake in the prevention and treatment of T1DM, as well as the underlying possible metabolomic (lipidomics) changes.

## 1 Introduction

Type 1 diabetes mellitus (T1DM) is an autoimmune disease characterized by the destruction of pancreatic β-cells, and encompasses 5–10% of total diabetes cases (Skyler, 2007; Lehuen et al., 2010; Wållberg and Cooke, 2013). The development of T1DM depends on at least three significant factors: genetic predisposition, environmental factors, and autoimmune response that results in β-cell destruction (Esposito et al., 2019).

Currently, the management of T1DM is based on insulin replacement therapy, with little focus on avoiding the onset and progression of the disease, which could be achieved by preventing β-cell damage *via* the modulation of cytokine response and the production of lipid mediators (Bellenger et al., 2011). The role of diet in the prevention or treatment of T1DM is poorly understood. There are some results suggesting that long-term consumption of polyunsaturated fatty acids (PUFAs), especially omega-3 (ω-3) PUFAs, could be beneficial in managing and preventing T1DM. Also, maternal exposure *via* breast milk can modify the immune response to islets and may affect the incidence of T1DM and the pathogenic course of the disease (Radzikowska et al., 2019).

In the current review, we focused on the current understanding of the beneficial effects of ω-3 PUFAs intake in the prevention and treatment of T1DM, as well as the underlying possible metabolomic changes. The aim of our paper was to present an overview of the molecular mechanisms and signaling pathways affected by ω-3 PUFAs and some of ω-3 PUFAs' main metabolites. Further, we reviewed the most recent preclinical reports and findings in human clinical and epidemiological studies, also touching on their metabolism, dietary recommendations, and key metabolomics aspects.

## 2 Methodology

A survey of the literature was performed using PubMed in order to find the most relevant articles reporting preclinical and clinical effects of ω-3 PUFAs in T1DM. Articles were limited to those published in the English language, focusing on the most recent works between 2010 and 2021 (62% of the cited material) but not neglecting any older relevant materials. For preclinical studies, the keywords and MeSH terms used were: “type 1 diabetes” and “omega 3”, “n-3 polyunsaturated fatty acids” and “mice”, “rats”, “animal model” or “cellular effects”, “pathways”, “signaling”. A total of 28 papers were selected after eligibility analysis, cross-checking and removing duplicates. For clinical trials, the used the keywords and MeSH terms were: “omega 3”, “n-3 polyunsaturated fatty acids” and “diabetes mellitus” and “type 1 diabetes mellitus”. A total of 65 papers were selected after eligibility analysis and cross-checking.

## 3 Pathogenesis of T1DM

T1DM is considered to be, in most cases, an autoimmune disease, and the immune-mediated attacks of T cells on pancreatic islets seem to be the root cause of its development. Typically, 85–90% of T1DM patients develop characteristic anti-insulin antibodies, antibodies against 65 kDa glutamic acid decarboxylase (GAD65), insulinoma-associated protein 2 (1A-2), and zinc transporter 8 (ZNT8). These antibodies can be used as diagnostic biomarkers for individuals with a high risk of developing T1DM (Das, 2017). However, only in a smaller percent of T1DM patients, no immune responses or autoantibodies are detected, and the reason for β-cell destruction is unknown. This is known as idiopathic T1DM, which has a strong genetic component (Katsarou et al., 2017). Genetic susceptibility can trigger the onset of T1DM, as more than 95% of T1DM patients carry predisposing alleles in the human leukocyte antigen (HLA) class II region, particularly specific DQ alleles (*HLA-DQ*) (Donath et al., 2003). Aside from the genetic predisposition, environmental factors, such as an imbalanced diet, or exposure to toxins, can heavily influence the development of T1DM (Bi et al., 2017; Petrakis et al., 2017).

T1DM progresses sequentially, and its three distinct stages are easily identifiable. The first stage is characterized by the presence of autoantibodies in normoglycemic individuals, while stage 2 involves the presence of autoantibodies and dysglycemia. In the last stage (stage 3), the well-known clinical signs and symptoms of T1DM become evident (Katsarou et al., 2017). The first autoantibodies (insulin or GAD65) are usually detected after 6 months of age, and the peak incidence of insulin autoantibody development is at 1–2 years old, mainly in individuals with the *HLA-DR4-DQ8* haplotype (Katsarou et al., 2017). This information is crucial as a balanced maternal diet during pregnancy or later on, during breastfeeding, may protect against the development of insulin autoantibodies (Bonifacio et al., 2008). Furthermore, the appearance of IA-2 autoantibody as a second or third autoantibody markedly increases the risk of the individual progressing to the third stage of disease (Katsarou et al., 2017).

In T1DM, the immune-mediated response starts with the interaction between B cells with an antigen, specifically via cell surface receptors. Then, activated B cells interact with CD4^+^ and CD8^+^ T cells and dendritic cells (DCs). DCs process β-cells autoantigens in lysosomal vesicles and transfer peptide fragments of the autoantigen to major histocompatibility complex (MHC) class II molecule. Those migrate to the plasma membrane and present the autoantigen to T helper cells (Th0) (Nicholas et al., 2011).

As the activated T cells recognize post-translationally modified peptides from pancreatic β-cells, this suggests that the loss of tolerance to derived autoantigens results from alterations in β-cells proteins. Finally, the specific activated T cells induce β-cell death (Katsarou et al., 2017).

DCs are involved first in the initiation of insulitis and later in the development of chronic inflammation observed in T1DM. DCs stimulate pro-inflammatory interleukin (IL)-12 secretion, inducing the transformation of Th cells to autoreactive effector T helper cells (Th1). Th1 cells induce a high production of pro-inflammatory cytokines such as IL-2 and interferon-gamma (IFN-γ), stimulate reactive oxygen species (ROS) generation and other inflammatory mediators, thus inducing pancreatic islet inflammation (insulitis) (Nicholas et al., 2011; Das, 2017).

As we mentioned before, numerous environmental factors may influence the development of T1DM, but it is unknown which one is the most important. For example, the intake of fat and protein from milk products was positively associated with the risk of advanced β-cell autoimmunity in children with *HLA-DQB1* (Virtanen et al., 2012). Also, viral infections or gestation events are proposed as possible contributors (Katsarou et al., 2017). Several factors, such as balanced maternal diet during pregnancy, breastfeeding, and vitamin D intake during infancy and/or childhood, are beneficial and have been associated with protective effects (Virtanen, 2016).

The successful management of T1DM includes glucose monitoring, insulin replacement therapy, and a healthy lifestyle. The regeneration of pancreatic islets and blockade of autoimmune attacks constitute further goals for the treatment of T1DM (Bi et al., 2017). Some scientific reports suggest that long-term consumption of PUFAs, especially ω-3 PUFAs, could be beneficial in the management and prevention of T1DM (Stene et al., 2003; Norris et al., 2007; Li et al., 2019).

## 4 Exposure and Daily Recommendations for ω-3 PUFAs Intake

In the general category of ω-3 PUFAs, the following compounds are found: α-linolenic acid (ALA; 18:3 ω-3), stearidonic acid (SDA; 18:4 ω-3), eicosapentaenoic acid (EPA; 20:5 ω-3), docosapentaenoic acid (DPA; 22:5 ω-3), and docosahexaenoic acid (DHA; 22:6 ω-3). These, along with the omega-6 (ω-6) PUFAs, are essential dietary compounds, as they are not synthesized in the human body, although they are fundamental components of phospholipids from cellular and subcellular membranes (Burdge and Calder, 2015).

ALA is typically consumed in the human diet via products derived from plant sources (seeds and derived products), as only plants biosynthesize it (Calder, 2011). The others ω-3 PUFAs, like DPA, EPA, and DHA, can be found in different proportions in fatty fish and other seafood or derived products, such as cod or tuna liver oil. For example, 1 g of fish oil contains up to 30% EPA and DHA (Margină et al., 2020a).

The current recommendations for ω-3 PUFAs intake are applicable only for healthy population groups (adults, pregnant women, children) or for patients with a specific disease, like high, and extremely high fasting-triglyceride levels (Kris-Etherton et al., 2002). To our knowledge, there is no recommended daily intake of ω-3 PUFAs for patients with T1DM.

The recommended European Food Safety Authority (EFSA) daily intake of ω-3 PUFAs (EPA and DHA) for adolescents and adults corresponds to 250 mg (in males and non-pregnant females) and increases with 100–200 mg DHA during pregnancy [Efsa Panel on Dietetic Products and Allergies (NDA), 2010]. The recommended intake by Food and Agriculture Organization (FAO) and World Health Organization (WHO) during pregnancy are higher (300 mg/day EPA and DHA), while in France or Belgium, national regulations stipulate a higher amount, of up to 500 mg/day (Sioen et al., 2017). The upper daily acceptable value for EPA and DHA was set at 2,000 mg for healthy adults (Rangel-Huerta et al., 2012).

The EFSA daily recommendation in children under 2 years old corresponds to 100 mg DHA [Efsa Panel on Dietetic Products and Allergies (NDA), 2010]. The FAO/WHO recommendations for EPA and DHA daily intake correspond to 10–12 mg/kg body weight for children of 6–24 months, 100–150 mg/day for children between 2 and 4 years old, and up to 250 mg/day for children between 6 and 10 years old (Nations FaAOotU, 2010). Higher national recommendations are applicable in other countries, like Belgium and France, where for children between 3 and 18 years, the recommended intake value for EPA and DHA is up to 500 mg/day (Sioen et al., 2017; Guesnet et al., 2019). Interestingly, in the United States, there are no specific recommendations for EPA or DHA in children. The daily recommendations exist for only ALA, considered the only essential ω-3 PUFAs. For children less than 3 years, adequate ALA intake corresponds to 0.7 g/day, while for children of 4–8 years of age, ALA recommendation is up to 0.9 g/day (Trumbo et al., 2002).

T1DM is the most common form of diabetes in children, and the incidence rate is increasing worldwide (Katsarou et al., 2017). Recent metabolomic studies suggested that an altered fatty acid profile in early life, which is partially connected with the ω-3 PUFAs intake, may signal the risk for islet autoimmunity (Niinistö et al., 2021). Moreover, neonates of T1DM mothers had lower levels of DHA and other fatty acids in plasma compared to babies of non-diabetic mothers (Ghebremeskel et al., 2004). Therefore, it is relevant to monitor the ω-3 PUFAs intake during early life, including during pregnancy and the first years of life. Only a few studies reported the average daily intake of total ω-6 and ω-3 PUFAs in the pediatric populations from different countries. The daily total PUFAs exposure and ω-6/ω-3 ratio were even estimated through food frequency questionnaires (FFQ) or calculated using the plasmatic levels of EPA, DHA, or other PUFAs.

In Spain, the EPA and DHA intake from fish and seafood was estimated in children between 6 and 12 years and adolescents in a cross-sectional study using FFQ. The mean estimated ingestion of EPA and DHA in the diet was 101.6 ± 5.9 mg/day, with no significant differences depending on gender. A significantly lower intake was observed in children (84.9 mg/day) than in adolescents (110.4 mg/day). These values represent only 50–60% of the EFSA recommended daily intake for this subpopulation (Martínez-Martínez et al., 2020). Comparable results are obtained in French children and adolescents in a national INCA 2 survey performed in 2006 and 2007, based on FFQ. 80% of the children and 90% of the adolescents ingested low quantities of DHA and EPA. Less than 25% of the children population consumed more than 125 mg/day DHA and 250 mg/day EPA and DHA. In adolescents, EPA and DHA intakes were between 144 and 184 mg/day, significantly lower than the national recommended value of 500 mg/day (Guesnet et al., 2019). Also, the average daily intakes of DHA and EPA in 7-year-old children from the United Kingdom, assessed through FFQ, were 49.7 mg/day and 35.7 mg/day, respectively. The ω-6/ω-3 ratio corresponds to 7.9:1, indicating a deficient intake of ω-3 PUFAs (Buckland et al., 2021).

Direct quantification of PUFAs in food samples was used to calculate the PUFAs intakes in the Canadian children aged 4–8 years. The results showed an intake of 38.4 mg/day EPA, 54.1 mg/day DHA, 38.4 mg/day DPA and 1,161 mg/day ALA. These values indicate a moderate gap for the ω-3 PUFAs intake (Madden et al., 2009). In a cross-sectional study conducted on United States children of 12–60 months old, ω-3 PUFAs intake did not change significantly with age, but ω-6 PUFAs intake increased with age (46% higher among children 49–60 months compared to 12–24 months). DHA intake was significantly low and constant across age groups (approx. 20 mg/day), while EPA intake significantly increased with age (Keim and Branum, 2015).

So, all published studies reported in children a low intake of ω-3 PUFAs and a corresponding imbalance between ω-6 and ω-3 PUFAs. Less information is available regarding newborns or early childhood. Based on the food consumption and complementary feeding, Schwartz et al. reported that ω-3 PUFAs intake during the first year of life is predominantly provided by breast milk and formula but, unfortunately, decreases continuously with increasing age, while the ω-6/ω-3 ratio increases (Schwartz et al., 2010). For newborns, maternal ω-3 PUFAs intake is relevant. For example, in French pregnant and lactating women, the mean estimated DHA intake based on FFQ was significantly low (155 mg/day in pregnant women and 185 mg/day in lactating women). In some cases, the ingested quantities of DHA were ten times lower than the national recommended value of 250 mg/day (Tressou et al., 2019).

It is essential to highlight that most published studies estimated the total PUFAs daily intake through food frequency questionnaires (FFQ), and just a few measured the plasmatic levels of EPA, DHA, or other PUFAs. This approach has several limitations, as the daily uptake is lower compared with the estimated intake. Also, in preterm or term infants, ω-3 PUFAs are poorly digested and absorbed due to the immaturity of the gastrointestinal system, exocrine pancreatic insufficiency or low immunoglobulins plasmatic levels (Martin et al., 2016; Pierzynowska et al., 2020). The bioavailability of ω-3 PUFAs is variable, depends on dietary sources and the form in which they exist (Shahidi and Ambigaipalan, 2018).

As seen above, the daily recommended ω-3 PUFAs intake varies slightly from country to country, mainly considering healthy adults, children, and pregnant women, with little regard to specific pathological conditions, an issue that should be addressed. Moreover, considering the possible links between an altered fatty acids profile in early childhood and T1DM, more attention should be given to the daily intake of ω-3 PUFAs in infants and pregnant/lactating women and the form in which they exist (free acids or esters).

## 5 Metabolism of ω-3 PUFAs

The biological effects exerted by ω-3 PUFAs are generated either by the parent compounds or their bioactive metabolites such as protectins, resolvins, and maresins. The ω-3 PUFAs can be released from phospholipids under the action of phospholipase A2 (PLA2) and constitute substrates for cyclooxygenases (COXs) and lipoxygenases (LOXs), which transform them into eicosanoids. Changing the relative content of PUFAs in the cell membranes (increasing the content of ω-3 wilst decreasing the ω-6 level) leads to an increase of anti-inflammatory metabolites generation (as a results of COXs and LOXs action on ω-3 and less on ω-6 acids) (Margină et al., 2020a; Margină et al., 2020b). Also, the leukotrienes (LTs) generated from EPA (5-series) exert weaker pro-inflammatory and vasoconstrictive actions compared to those obtained from ω-6 (4-series, obtained from arachidonic acid) (De Caterina et al., 2007).

**FIGURE 1:**
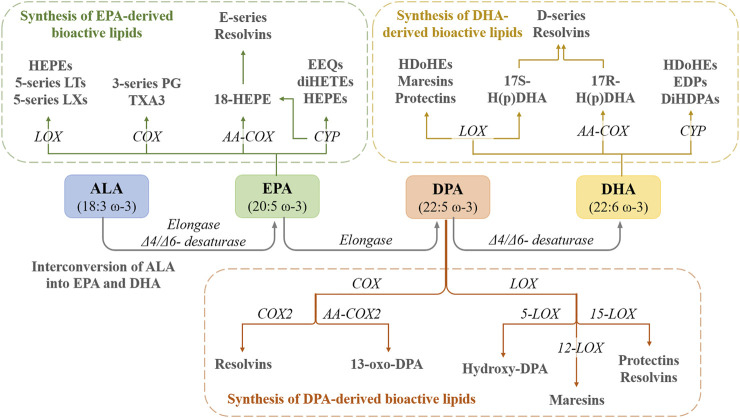
The interconversion of ALA into EPA and DHA in the human body as well as the conversion of these PUFAs into active metabolites. HEPEs—hydroxyeicosapentaenoic acids; LTs—leukotrienes; LXs—lipoxins; LOX—lipooxigenase; PG—prostaglandin; TXA3—thromboxane A3; COX—cyclooxygenase; AA-COX—aspirin-acetylated COX; EEQs—epoxyeicosatetraenoic acids; diHETEs—dihydroxyeicosatetraenoic acids; CYP—cytochrome P450; HDoHEs—hydroxydocosahexaenoic acids; H(p)DHA—hydroperoxydocosahexaenoic acid; EDPs—epoxydocosapentaenoic acids; DiHPAs—dihydroxydocosapentaenoic acids.

In the human body, ALA can be transformed, under the action of Δ6-desaturase, elongase, and Δ5-desaturase into EPA, which can be converted into DHA, potential substrates for COXs and LOXs (De Caterina et al., 2007). The rate of biotransformation from ALA to EPA and DHA is affected by several factors such as age, sex, disease, or genetics (Baker et al., 2016) and by ω-6 PUFAs intake. A high intake of ω-6 PUFAs inhibits the endogenous conversion of ALA to EPA and DHA, thus inducing a deficiency state of ω-3 PUFAs, by reducing the activity of desaturase enzymes (Robertson, 1988; Storlien et al., 1991; Raheja et al., 1993) (Figure 1).

Recently, the lipidomic profiling of free fatty acids in T1DM patients highlighted a specific alteration in lipid metabolism, particularly the elongase index (calculated as the oleate: palmitoleate ratio), which was increased, while the 5-desaturase index was unchanged, compared to the control group (Sobczak et al., 2021). These changes indicate that T1DM may partially influence the elongase activity and thus the metabolic profile of PUFAs.

EPA can be metabolized via the COX pathway into 3-series prostaglandins (PG) and thromboxanes (TXA3), or by aspirin-acetylated COX-2 to 18-hydroxyeicosapentaenoic acid (18-HEPE), and then to E-series Resolvins (RvEs) through the LOX pathway. EPA is also biotransformed into HEPEs and 5-series leukotrienes (LTs) and 5-series lipoxins (LXs) and by the LOX pathway. Furthermore, EPA is a substrate for cytochrome P450 and biotransformed into epoxyeicosatetraenoic acids (EEQs) and dihydroxyeicosatetraenoic acids (diHETEs) (Duan et al., 2021) (Figure 1).

The D-series of Resolvins (RvDs; D1-D6), Maresins (MaR1, MaR2), and Protectins (PDs) are derived from DHA *via* LOX pathway (Calder, 2015), while DPA (docusapentaenoic acid) is biotransformed into PDs, MaR1-MaR3, and RvDs (D1, D2, and D5) through LOX pathway and into 13-series Rvs though COX pathway (Duan et al., 2021). Furthermore, cyclic oxygenated metabolites of ω-3 PUFAs were identified, such as phytoprostanes F3-isoprostanes and F4-neuroprostanes, some with notable beneficial effects. For example, EPA and DHA-related isoprostanes exhibited *in vitro* potent anti-inflammatory effects linked to the inhibition of the NF-κB (nuclear factor kappa-light-chain-enhancer of activated B cells) and Nrf2 (nuclear factor erythroid 2-related factor 2) pathways (Galano et al., 2015).

The biosynthesis of RvDs is increased when the diet is supplemented with ω-3 PUFAs. In obese women treated for 3 months with a daily supplement of 1.8 g EPA and DHA, DHA-derived resolvins production was stimulated (RvD1 and RvD2 > 60 pg/ml plasma) (Polus et al., 2016). The levels of ω-3 PUFAs metabolites are also influenced by age or associated pathologies (Calder, 2020). For example, in patients with type 2 diabetes (T2DM), the RvE1 level was decreased compared with the healthy subjects, while RvE2 and RvE3 were below detection limits (Freire et al., 2017).

## 6 The Cellular Mechanisms of Action of ω-3 PUFAs in T1DM

At least two major mechanisms of action can explain the beneficial effects of ω-3 PUFAs in T1DM: anti-inflammatory action and the inhibition of autoimmunity. It is known that inflammation is a paramount component of T1DM contributing to β-cells dysfunction and subsequent apoptosis. In this regard, the fat-1 transgenic mouse model provided compelling evidence to support ω-3 PUFAs anti-inflammatory action. The supplementary, non-obese diabetic (NOD) mice model provides information on the potential of ω-3 PUFAs in suppressing T1DM autoimmunity.

There are several molecular targets for the anti-inflammatory effects of ω-3 PUFAs, such as G-protein coupled receptor 120 (GPR120) and peroxisome proliferator-activated receptor-gamma (PPARγ) (Im, 2012). Oh, et al. reported that at high concentrations, ω-3 PUFAs (EPA and DHA) activate GPR120, which couples to β-arrestin 2, inhibits TAB1-mediated activation of TAK1, and suppress NF-κB activation and macrophage-mediated inflammatory responses (Oh et al., 2010). PPARγ activation prevents NF-κB nuclear translocation and reduces inflammatory responses, and this mechanism could partially explain ω-3 PUFAs’ anti-inflammatory effects. ALA, DHA, and EPA are weaker agonists of PPARγ, while protectin D1 is much more potent (Yamamoto et al., 2005). Further, ω-3 PUFAs also interfere with membrane activation of NF-kB by binding to Toll-like receptor 4 (TLR-4) (Im, 2012), while other PUFAs’ metabolites bind strongly to different receptors. RvD1 specifically interacts with GPR32 and formyl peptide receptor 2 (ALX/FPR2) (Krishnamoorthy et al., 2010), while RvE1 binds to LTB4 receptor BLT1 and Chem R23 and reduces IL-12 production and inflammation (Arita et al., 2007).

Some *in vitro* studies assessed the effects of ω-3 PUFAs and their bioactive metabolites in human and animal pancreatic islets and evaluated the direct impact on the function and viability of pancreatic β-cells. *In vitro* treatment of human islets with RvE1 (500 nM for 24 h) prevented LPS-induced increase in mRNA and levels of pro-inflammatory markers IL-8 and monocyte chemoattractant protein-1 (MCP-1), but did not affect insulin secretion. RvE1 counteracted the apoptotic effect of IL-1β, IFN-γ, and TNF-α on islet membrane integrity (Lund et al., 2010).

In isolated islets derived from fat-1 transgenic mice, the cytokine-induced activation of NF-κB and extracellular signal-related kinase 1/2 (ERK 1/2) were attenuated, while the mRNA levels of pancreatic and duodenal homeobox 1 (PDX-1) and insulin were significantly increased compared with the wild-type islets. Moreover, the islets strongly resisted cytokine-induced cell death (13 *vs.* 8% in the wild-type islets) (Wei et al., 2010). *Ex vivo* DHA treatment of peritoneal macrophages from mice with streptozotocin (STZ)-induced T1DM resulted in reversing the diabetes-induced changes, as increased pro-inflammatory cytokine/chemokine levels, nitric oxide (NO) secretion, NLRP3 and inducible NO synthase (iNOS) protein levels (Davanso et al., 2020).

NOD mice model was also useful to investigate ω-3 PUFAs mechanism of action as spontaneously develop T1DM in a manner that reproduces the human disease (Anderson and Bluestone, 2005; Shoda et al., 2005). In the NOD model, dietary intervention with ω-3 PUFAs resulted in the modulation of the differentiation of T helper (Th, CD4^+^) cells and regulatory T cells (Tregs), also alleviating the inflammatory burden by reducing IFN-γ, IL-17, IL-6, and TNF-α levels (Bi et al., 2017). Similar effects were reported on the differentiation of Th cells isolated from human peripheral blood mononuclear cells (PBMCs) by reducing the Th1 cells population and balancing the Th1/Th2 ratio and suppressing IL-17A production and significantly increasing IL-4 and IL-10 secretion. The effects are the results of mammalian target of rapamycin (mTOR) complex 1 (mTORC1) inhibition. In contrast, the ω-6 PUFAs (such as arachidonic acid) increased the number of Th17 cells, without significant effect on Treg numbers, and drastically exacerbated Th1/Th2 cells ratio (Bi et al., 2017). So, mTOR signaling has emerged as an essential pathway *via* which ω-3 PUFA regulates the differentiation of T lymphocytes (Chi, 2012).

**FIGURE 2:**
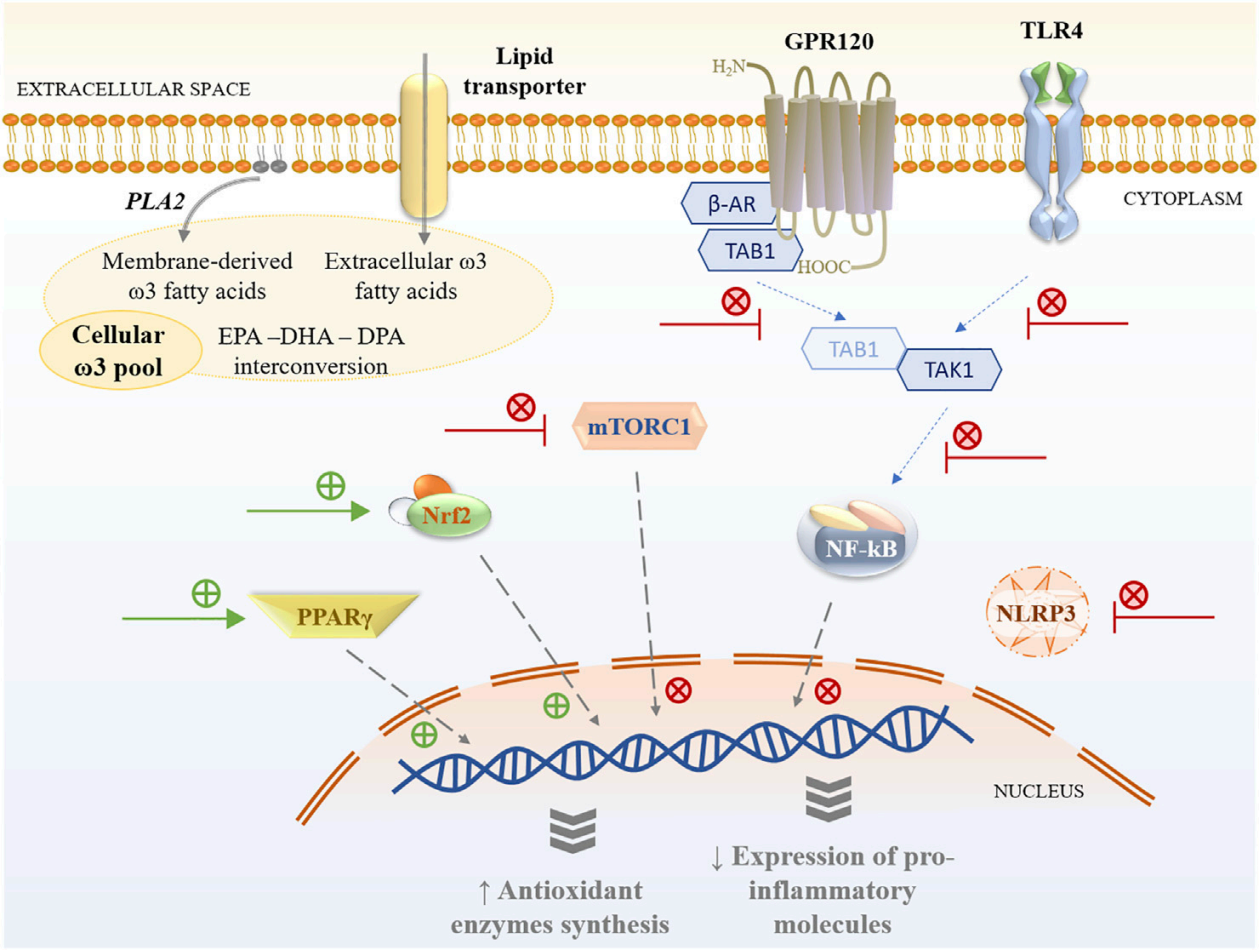
Cellular pathways modulated by ω-3 PUFAs and their metabolites. GPR120—G-protein coupled receptor 120; TLR4—Toll-like receptor 4; PLA2—phospholipase A2; β-AR—β; TAB1—Mitogen-activated protein kinase 7-interacting protein 1; TAK1—Mitogen-activated protein kinase 7; mTORC1—mammalian target of rapamycin complex 1; Nrf2—Nuclear factor erythroid 2-related factor 2; PPARγ—Peroxisome proliferator-activated receptor gamma; NF-κB–Nuclear factor kappa-light-chain-enhancer of activated B cells; NLRP3—NLR family pyrin domain containing 3.

The exposure of PBMCs collected from T1DM patients to ω-3 PUFAs (EPA and DHA) increased the antioxidant defense by enhancing reduced glutathione, SOD, and CAT activities and in modulating T cell functions and proliferation (Merzouk et al., 2008). Further, increasing ω-3 PUFAs levels (*via* supplementation or stimulating endogenous synthesis/interconversion) blocked the development of autoimmunity, prevented lymphocyte infiltration into regenerated islets, and sharply elevated the expression of the β-cell markers—PDX1 and paired box 4 (Pax4) (Bi et al., 2017) (Figure 2).

## 7 Preclinical Evidence Regarding the Role of ω-3 PUFAs in Islet Autoimmunity, Inflammation, and β-cell Function

In the fat-1 transgenic mouse model, in which endogenous production of ω-3 PUFAs is achieved through overexpressing a *C*. *elegans* ω-3 fatty acid desaturase gene—mfat-1, the islets cells contained higher levels of ω-3 PUFAs and lower levels of ω-6 PUFAs than non-transgenic cells (Kang, 2007). The transgenic islets were resistant to cytokine-induced cell death when challenged with IL-1β, IFN-γ, and TNF-α. In addition, the cytokine-induced activation of NF-κB and ERK1/2 was decreased, while activation of PDX-1, glucokinase, and insulin-1 was increased (Wei et al., 2010). These findings are consistent with other results derived from the same fat-1 transgenic model (Li et al., 2014), as decreased tissue levels of PGE2, LTB4, and MCP-1 were observed post-high-fat diet regimen compared to non-transgenic animals. Hepatic TNF-α-induced NF-κB signaling was significantly reduced, while PPARγ was stimulated leading to insulin sensitivity and signaling improvement and inflammation attenuation (Li et al., 2014). Furthermore, post-STZ administration, fat-1 mice did not develop hyperglycemia or exhibit β-cell destruction and increased levels of pro-inflammatory molecules (TNF-α, IL-1β, iNOS) compared with wild-type mice. In addition, they had large pancreatic islets and normal insulin level similar to the one observed in vehicle-treated animals (Bellenger et al., 2011).

The authors also investigated PUFA-derived metabolites and observed that in the fat-1 mice, PGE2 and 12-HEPE were decreased, while the anti-inflammatory lipoxin A4 was detected. However, only 18-HEPE, a precursor of the RvE1, was significantly increased in STZ-induced fat-1 mice. The protective effects against the development of T1DM were correlated with the downregulation of NF-κB p65 subunit expression in the pancreatic tissue and the subsequent decrease of pro-inflammatory cytokines synthesis (Bellenger et al., 2011). Another study linked the protective effects against the STZ-induces damage to an upregulated autophagic activity in β-cells (Hwang et al., 2015).

Oral treatment of STZ- T1DM rats with 0.5 ml kg/day cod liver oil (containing mainly EPA and DHA), for 12 weeks decreased lipid peroxidation products as MDA level, normalized catalase and glutathione peroxidase activities, and partially controlled hyperglycemia in diabetic animals (Hünkar et al., 2002). Some researchers observed that pre- and simultaneous treatment with EPA and DHA-rich concentrated fish oils protect the animals against alloxan-induced T1DM, resulting in significantly decreased levels of plasma glucose and increased levels of insulin, impeding the development of the disease.

The treatment with EPA and DHA-rich oils also restored the altered antioxidant status (Krishna Mohan and Das, 2001). In contrast, other studies suggest that only pre-treatment (5 days prior to alloxan administration) with EPA and DHA, GLA, and AA is effective and protects the animals against alloxan effects (Suresh and Das, 2001).

There are also some mechanistic studies that investigated the effects of RvD1 *in vivo*.

It was observed that in the corneal epithelium from the diabetic mice, RvD1 decreased oxidative stress, reducing NADPH expression, and ROS accumulation while restoring the diabetes-impaired Nrf2-ARE signaling along the epithelial regeneration/proliferation-related signaling pathways (p-EGFR, Sirt1, Ki67) (Zhang et al., 2018)**.** In an STZ-induced T1DM male Wistar model, RvD1, restored the altered plasma levels of TNF-α, IL-6, lipoxin A4, and brain-derived neurotrophic factor (BDNF) (Bathina and Das, 2021). Moreover, it exerted pancreatic antiapoptotic, anti-inflammatory, and antioxidant effects by restoring the expression of *COX-1/COX-2/PPARγ* genes along the downstream insulin signaling proteins Gsk-3β/FOXO1, and of *Bcl2/Pdx* gene modulating β-cell proliferation, also enhancing antioxidants and limiting the generation of lipid peroxides (Bathina and Das, 2021). Metabolomic analysis revealed that RvD1 and EPA-derived prostaglandin D3 levels were significantly increased in pancreatic samples of mice fed with EPA and DHA-enriched diet. As both ω-3 PUFAs metabolites strongly inhibited the differentiation into Th1 cells while promoting Th2 cell and Treg populations, it was suggested that they are at least partly responsible for balancing CD4^+^ T cell differentiation, which plays a crucial role in the development of autoimmunity in T1DM (Bi et al., 2017).

Kagohashi et al. reported that a lower ω-6/ω-3 ratio (3 versus 14.5) in the diet of female NOD mice during gestation and lactation significantly reduced the development of insulitis and delayed the onset of T1DM in susceptible offspring (Kagohashi et al., 2010). The same authors observed that this diet significantly prolonged NOD mice survival, with more remaining islets when administered within 6 days after the onset of overt diabetes. This effect was not observed when the dietary intervention occurred later than 9 days after the onset of overt diabetes (Kagohashi and Otani, 2010). In another study conducted on NOD mice, supplementation of the diet during pregnancy and weaning of offspring with vitamin D3 and/or fish-oil, corresponding to 2.9 g PUFAs/100 g (ω-6/ω-3 ratio 1:2) for 30 weeks, was not associated with a significant delay in the onset of diabetes in offspring (Schmid et al., 2004). Other researchers observed that feeding STZ-T1DM pregnant rats with ω-6/ω-3 ratio 0.49, induced beneficial effects such as a significant reduction of glucose levels, upregulated IL-4 mRNA expression in the spleen, and IL-10 mRNA in the pancreas, and spleen and significantly increased serum IL-4 concentrations. Furthermore, the incidence of macrosomia was decreased by ω-3 PUFAs diet compared to the standard diet. Moreover, ω-3 PUFAs diet diminished Th1 mRNA quantities and increased the levels of IL-4, but not of IL-10 in macrosomic offspring (Khan et al., 2006).

Bi et al. reported that daily intake of EPA and DHA (3.6 g/kg b.w., as fish oil) for 35 weeks in NOD mice reduced the incidence of T1DM, as only 33% of the treated mice were diabetic, compared with 80% from the control group. EPA and DHA significantly reduced the incidence of severe insulitis and elevated insulin secretion. The serum and tissue fatty acid profile analysis revealed significant changes with dietary supplementation (Bi et al., 2017).

**FIGURE 3:**
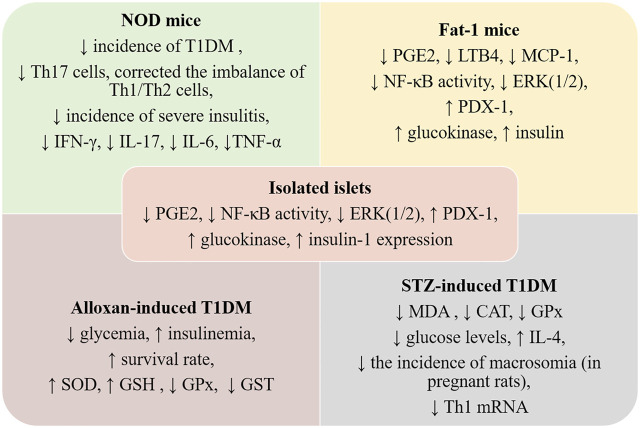
Preclinical data regarding ω-3 PUFAs intake and TD1M. NOD—non-obese diabetic; Fat-1 mice—murine model overexpressing a *C*. *elegans* ω-3 fatty acid desaturase gene—mfat-1; LTB4—leucotriene B4, MCP-1—monocyte chemoattractant protein-1; ERK (1/2)—extracellular signal-related kinase 1/2; PDX1—pancreatic duodenal hemeobox-1; GSH—glutathione; GPx—glutathione peroxidase; CAT—catalase; IL—interleukin; MDA—malonyldialdehyde; SOD—superoxid dismutase; GST—glutathione transferase; PGE2—prostaglandin E2; NF-kB—nuclear factor kappa-light-chain-enhancer of activated B cells; Nrf2—nuclear factor erythroid 2-related factor 2; Th—T helper cells.

As seen above, several animal studies investigated the therapeutic potential of ω-3 PUFAs, reporting their potency in suppressing autoimmunity, protecting and enhancing islet functions, and inducing anti-inflammatory, antioxidant, and antiapoptotic effects. These studies were conducted in the chemical-induced diabetic model (STZ/alloxan-induced diabetic rats), in NOD mice, or the fat-1 transgenic mouse model. All are relevant animal models widely used to investigate T1DM, while NOD and fat-1 mice models are also used for metabolomic studies (Anderson and Bluestone, 2005; Alcazar et al., 2019). The outcomes of these studies are summarized in Figure 3.

## 8 Clinical Data Arguing the Effects Induced by ω-3 PUFAs in T1DM

Data regarding the effects of ω-3 PUFAs are incredibly vast but equally controversial, with many researchers arguing their health benefits and others claiming the absence of these effects. This type of heterogeneity of reports is also found when looking into their effects in either TD1M or T2DM (Shahidi and Ambigaipalan, 2018).

A study by Djoussé et al. published in 2011 (Djoussé et al., 2011) reported a positive association of high levels of ω-3 PUFAs (≥0.20 g/day or ≥ two servings of fish/day) with the risk of T2DM; other authors came to the same conclusion (Østerud and Elvevoll, 2011) and attributed this correlation to the fact that a high concentration of PUFAs in cell membranes makes them prone to oxidation and a higher level of oxidation end-products induces inflammation, both mechanisms (oxidative stress and inflammation) being involved in the development of T2DM. On the other hand, a review of literature data performed in 2004 (MacLean et al., 2004), including diabetes as well as metabolic syndrome patients, suggested the inability of ω-3 PUFAs to have a direct outcome on fasting plasma glucose, insulin level, glycosylated hemoglobin (HbA1C) levels or insulin sensitivity. Another review published in 2014 (Schwab et al., 2014) found that the evidence of an association between dietary ω-3 PUFAs and T2DM was inconclusive, even if there was limited-suggestive evidence of an inverse correlation between ω-3 PUFAs intake from fish and the risk of T2DM. Others report the opposite—that ω-3 PUFAs-supplemented diets are strongly associated with protective effects against T2DM (Wang et al., 2003), in association with increased insulin sensitivity and a decrease of C-reactive protein (CRP) (Tsitouras et al., 2008).

Some epidemiological data support the theory that the deficit of ω-3 PUFAs, as well as an imbalance of ω-6/ω-3 ratio, could contribute to the activation of immune cells and their consequent attack on pancreatic β-cells (Nathan, 1987; Rabinovitch et al., 1990). The incidence of T1DM is much higher in formula-fed babies compared to breastfed ones, this correlating with the fact that breast milk is rich in long-chain ω-3 PUFAs; also, the incidence of T1DM is constantly rising in adolescents and young people from industrialized countries, since their diets are based on junk food rich in saturated fatty acids, as well as ω-6 PUFAs (Borch-Johnsen et al., 1984; Simopoulos, 1991).

In the United States number of DM cases sharply increased after 1960, mirroring the increased use of ω-6 rich seed oils after issuing reports regarding their ability to lower plasma cholesterol levels in the general population (Harris et al., 1998). In contrast, epidemiological data show that Eskimos, with a diet rich in ω-3 PUFAs and characterized by an optimum ω-6/ω-3 ratio, have a lower prevalence of autoimmune diseases, including DM, compared to Western societies, characterized by imbalanced diets concerning the fatty acids intake and ratios (De Caterina et al., 2007). In a group of elderly Dutch subjects, a strong inverse correlation between dietary fish intake and diabetes risk/glucose intolerance was found (Feskens et al., 1995). Further, while an important intake of PUFAs is characteristic of the Indian traditional diet, European immigrants from India generally change their diet, increasing the ω-6/ω-3 ratio. This is mirrored by a significant increase in insulin-dependent diabetes cases while lowering the omegas’ ratio reverses the epidemiological situation (Raheja et al., 1993; De Caterina et al., 2007).

Recently, the European Prospective Investigation into Cancer and Nutrition (EPIC)-InterAct case-cohort study conducted on 11,247 incident cases of adult-onset diabetes reported that high fish intake or relatively high ω-3 PUFA plasma levels may partially counteract the increased incidence of T1DM in individuals who are GAD65 antibody positive (Löfvenborg et al., 2021).

There are also studies on T1DM patients. For example, in 1986, Haines et al. administered to 41 T1DM patients either 15 g fish oil or 0.6 g olive oil for 6 weeks and observed a decrease in platelet thromboxane synthesis, an increase in LDL level, but no effects on the HbA1C (Rose et al., 1976; Haines et al., 1986). So, one of the beneficial effects associated with ω-3 PUFAs intake in T1DM patients was the decrease of platelet reactivity, even if no changes in other coagulation parameters were reported (Haines et al., 1986; Miller et al., 1987; Rillaerts et al., 1989; Landgraf-Leurs et al., 1990). Results were later reproduced in other small studies that pointed out that ω-3 rich fish oil supplementation had no significant effect on the glycemic balance in insulin-dependent patients (Miller et al., 1987; Tilvis et al., 1987).

Another study observed that cod liver oil, a well-established source of ω-3 PUFAs improved the blood pressure, but not HbA1C, in insulin-dependent patients with impaired kidney function, indicating that fatty acids can be more effective in diabetes patients with associated hypertension (Jensen et al., 1989; Simopoulos et al., 1991). Results regarding the effect on microalbuminuria associated with T1DM are variable; one small study (5 patients) reported a decrease of microalbuminuria after 1.8 g EPA/day administered for 6 months (Hamazaki et al., 1990), but these results were not reproduced by other larger studies (Haines et al., 1986; Lungershausen et al., 1997). In the case of 27 T1DM patients without preexisting microvascular complications, no beneficial effects of daily high-dose-bolus ω-3 PUFAs supplementation for 6-months on blood pressure, glycemic control, lipid profile, inflammation markers were observed in a randomized, double-blind, placebo-controlled trial (O’Mahoney et al., 2020).

Even if no significant effects were reported for glycemic control, all these studies described that 2–4 g ω-3 PUFAs/day leads to an improvement of the cardiovascular risk in T1DM patients, especially through the reduction of hypertriglyceridemia (Harris, 1997; Krauss et al., 2000). Results concerning the effect on low-density lipoprotein (LDL) are inconsistent; some of the clinical studies observed an increase in LDL probably associated with smaller particles, with lower triglycerides content and less atherogenic capacity (less prone to oxidation) (Mori et al., 1988; Stacpoole et al., 1989), while others report an increase of the same lipoproteins (Miller et al., 1987; Schmidt et al., 1989). Also, an increase of HDL was reported for T1DM patients receiving high doses of EPA (15 capsules of 1 g MaxEpa/day) for 3 weeks (Mori et al., 1991). The effects of ω-3 PUFAs supplementation (4.0 g/day) on the PBMC’s proliferative response and on the secretion of monokines and arachidonic acid metabolites from PBMC and monocytes from insulin-dependent diabetes mellitus, as well as healthy patients were also evaluated. Results showed that ω-3 PUFAs inhibited the proliferation of PBMC and reduced IL-1β immunoreactivity in PBMC and monocytes but did not alter monokine, PGE2 or LTB4, secretion in healthy or T1DM subjects (Molvig et al., 1991).

Another study evaluated the effect induced by 330 mg/day DHA and 630 mg/day EPA on the lipid composition of cell membrane and metabolic control, using 18 T1DM patients randomized to receive either supplementation or their usual diet. Results show that the increased concentration of ω-3 in the red blood cells membrane was correlated with a slight improvement of neural conduction and a significant decrease of HbA1C, not accompanied by changes in the dose of insulin required (Stiefel et al., 1999). Patients with T1DM received 750 mg EPA, 560 mg DPA, and 1,020 mg DHA for 1 year, and the effects on neuropathy were evaluated; results showed that ω-3 PUFAs supplementation supported early nerve regeneration in T1DM patients with a broad spectrum of nerve injury (Lewis et al., 2017).

There are also observational trials searching for preventive dietary factors correlated with the development of T1DM based on the immunological mechanism of the disease. For example, in a longitudinal, observational study—the Diabetes Autoimmunity Study in the Young (DAISY)—including 1,770 children at increased risk for T1DM searched for a direct association between the ω-3 or ω-6 intake and the development of islet autoimmunity (IA) in children. Results showed that the intake of ω-3 PUFAs increased ω-3 fatty acid content of erythrocyte membranes and is correlated with the decrease of IA risk in children with genetic risk factors for T1DM (Norris et al., 2007). In contrast, Miller observed that ω-3 and ω-6 fatty acid levels of erythrocyte membranes were not associated with conversion to T1DM in children with islet autoimmunity (Miller et al., 2011). The lack of evidence was explained by the relatively small size of cases included in this study.

The TrialNet Nutritional Intervention to Prevent (NIP) Type 1 Diabetes Pilot Trial also evaluated the impact of DHA on the risk of developing T1DM due to IA when is administered in the last trimester of pregnancy (if the child’s father or the child’s full or half-sibling had T1DM) and in the first few years of life (for 6 month or younger infants, whose mother, father, or full or half-sibling had T1DM). No direct effect on immunity was observed, even if the DHA plasma level, as well as the concentration in erythrocytes membranes, increased^1^ (Miller et al., 2011; Lewis et al., 2017). Also, results from a multicenter, two-arm, randomized, double-blind pilot trial evaluating the effects of DHA supplementation, either in the last trimester of pregnancy (41 newborns) or in the first 5 months after birth (57 infants), pointed out that the DHA level in red blood cells membrane increased and the inflammatory marker hsCRP decreased in the breastfed DHA-treated infants compared to all formula-fed infants at the age of 12 months. Moreover, the study leads to the conclusion that supplementation of infant diets with DHA is safe (Chase et al., 2015). Results concerning safety were confirmed by an open-label, 2:1 randomized study including 15 T1DM children receiving autologous umbilical cord blood infusion followed by 1 year of supplementation with vitamin D and DHA (Haller et al., 2013).

A study performed in Norway analyzed the level of EPA and DHA in serum samples of pregnant women, 89 whose babies developed T1DM and 125 whose children did not develop it by the age of 15. No significant association between DHA or EPA in maternal serum and risk of T1DM in the offspring was observed (Sørensen et al., 2012). These results are contradicted by Stene et al., who published the results obtained in a case-control study, also performed in Norway, including 545 cases of childhood-onset T1DM, and 1,668 control subjects, who received cod liver oil or other vitamin D supplements, either during pregnancy or during the first year of life of the infants. Results proved that cod liver oil, as the source of ω-3 PUFAs, in the first year of life was correlated with a significantly lower risk of T1DM, probably due to the anti-inflammatory mechanism, but vitamin D did not have any association with the risk of disease (Stene et al., 2003; Li et al., 2019). Another prospective randomized placebo-controlled study evaluating the effect of ω-3 PUFAs supplements on the risk of autoimmune insulitis generating T1DM; the trial included 47 pregnant T1DM women who received an enriched diabetic diet with EPA and DHA twice a day (616 mg DHA and 120 mg EPA) and 43 pregnant diabetic women on a standard diabetic diet with placebo. Results showed that the ω-3 PUFAs supplementation was associated with a steady increa1se of the C peptide level during the pregnancy (from the first to the third trimester) (Horvaticek et al., 2017).

## 9 Metabolomics, ω-3 PUFAs, and T1DM

Metabolomics, especially lipidomics, has gained significant prominence in diabetes research within the last few years, as it can assess certain changes in metabolites that are correlated with increased risk of diabetes (Arneth et al., 2019; Johnson et al., 2019). For example, the risk for developing T1DM or T2DM was positively correlated to the levels of aromatic amino acids as phenylalanine and branched-chain amino acids (BCAAs) (Ilonen et al., 2013; Törn et al., 2015; Bloomgarden, 2018).

A high dietary intake of BCAAs was reported to increase insulin resistance since high levels of BCAAs stimulate the mTORC1 pathway, resulting in activation of S6 kinase and inhibition of insulin receptor substrates through serine phosphorylation (Johnson et al., 2019). Additionally, defective BCAAs oxidative metabolism observed in individuals with obesity leads to the accumulation of BCAAs and toxic metabolites. The accumulation of metabolites promotes β-cell apoptosis and is associated with insulin resistance and T2DM (Lynch and Adams, 2014). Furthermore, T1DM and T2DM development is associated with lipidomic changes, such as plasma phospholipids, triglycerides, sphingolipids, and glycerophospholipids (Cheng et al., 2012; Wang-Sattler et al., 2012; Mahendran et al., 2013). Also, levels of pentadecanoic acid, heptadecanoic acid, stearic acid, and conjugated linoleic acid are higher in individuals with T1DM (Hakola et al., 2021). Other studies in children carrying *HLA-DQB1* confirmed that higher serum levels of EPA and DPA during infancy are associated with a lower risk of islet autoimmunity, while higher arachidonic: docosahexaenoic and ω-6/ω-3 ratios with an increased risk (Virtanen et al., 2010; Niinistö et al., 2017; Niinistö et al., 2021).

The fatty acids profile is advantageous to be assessed, as fatty acids are considered biomarkers of dietary exposure to ω-3 PUFAs. Furthermore, serum total fatty acids are a valuable marker of inflammation, useful in cases of chronic inflammation like autoimmune diseases, including T1DM (Tsoukalas et al., 2019a).

Recent metabolomic studies also suggested that altered serum fatty acid profile or abnormal lipid composition of red cells membrane in early life, which are signs of metabolic lipid dysregulation, may also signal the risk for islet autoimmunity (Virtanen et al., 2010; Niinistö et al., 2017; Tsoukalas et al., 2019b; Tsoukalas et al., 2020; Niinistö et al., 2021). As we mentioned before, in fat-1 mice, where the level of ω-3 PUFAs is increased, a higher concentration of EPA and DHA was observed in erythrocytes. In addition, mass spectrometry techniques revealed an increased level of metabolites, such as 18-HEPE and 17-HDHA, compared with wild-type animals (Gomolka et al., 2011). Comparable results on the composition of red-cell membranes were obtained in children at increased risk for T1DM (Norris et al., 2007; Skyler, 2013). The authors also suggested that only lower membrane levels of DPA were predictive of islet autoimmunity, while ALA, EPA, and DHA levels were not (Norris et al., 2014).

There are some data regarding the influence of ω-3 PUFAs supplementation on the plasma metabolome of healthy older adults. Quantitative metabolomics was performed in plasma using proton nuclear magnetic resonance (1H-NMR) and mass spectrometry. ω-3 PUFAs induced a reduction of triglycerides and very-low-density lipoprotein (VLDL), a modest increase HDL, and a shift in the composition of HDL subclasses. Further metabolite profiling revealed pronounced changes in phospholipids, cholesterol esters, diglycerides, and triglycerides. The majority were decreased in response to ω-3 PUFAs supplementation, apart from the individual species containing long-chain polyunsaturated acyl chains, which increased (Xyda et al., 2020). These results should be considered in conjunction with data on T1DM patients receiving ω-3 PUFAs (Harris, 1997; Krauss et al., 2000).

In healthy adults, ω-3 PUFAs supplementation reduced Δ6-desaturase index and increased Δ5-desaturase index, both activities being estimated from the circulation phospholipids fatty acids composition (Vessby et al., 2013). As a low Δ5-desaturase index is associated with insulin sensitivity, this marker can be used to investigate ω-3 PUFAs effects in patients with T1DM. Limited data is available on the metabolic changes induced by the combinations of ω-3 PUFAs with other nutrients in T1DM patients. For example, Balderas et al. used the capillary electrophoretic method to analyze the urine of diabetic children and to investigate the metabolic fingerprinting after 1-year supplementation with a combination of rosemary extracts and vitamin E of a diet with ω-6/ω-3 ratio lower than 4. The levels of some metabolites with the aromatic ring, such as phenyllactate and p-OH-phenyllactate, phenaceturate and benzoate were significantly changed due to treatment (Balderas et al., 2010).

## 10 Benefits of Using Metabolomics in T1DM Clinical Practice

There are no doubt that metabolomics has the potential to be an effective tool in clinical practice for the early diagnosis of metabolic diseases, or it could serve as a predictor of treatment response. It is already demonstrated that specific lipid changes precede islet autoimmunity and T1DM progression (Oresic et al., 2008; La Torre et al., 2013; Lamichhane et al., 2018). Therefore evaluation of the serum fatty acids ratios as part of metabolomics profiling can provide clinical input for the early identification of metabolic abnormalities before the onset of symptoms.

Also, the lipidomic analysis of erythrocytes’ membranes, especially in early life has a predictive value to identify the subjects who are at-risk for developing T1DM.

Furthermore, a distinct plasma amino acids pattern and fatty acids profile was identified in children with T1DM at the ages of 3 and 6 months, before the appearance of islet autoantibodies. Different metabolic pathways were dysregulated, including alanine, aspartate and glutamate metabolism, or linoleic acid, and AA metabolism. The appearance of islet cell autoantibodies was associated with decreased glutamic and aspartic acids (Lamichhane et al., 2019). Comparable results were previously reported in a cohort study in children who later progressed to T1DM. Using two-dimensional gas chromatography coupled to time of flight mass spectrometry (GC x GC-TOF/MS), glutamic acid was up to 32-fold increased, and BCAAs such as leucine and isoleucine were also increased before seroconversion. In contrast, ketoleucine concentration was diminished (Oresic et al., 2008).

Taken together, we suggest that in clinical practice, the assessment of plasma lipid profile or the lipidomic analysis of erythrocytes’ membranes together with the plasma amino acids pattern could be helpful in the early identification of the metabolic changes in T1DM and possible to follow disease progression. However, further studies including large-scale prospective studies, are needed to strengthen these claims and validate these biomarkers.

As lipidomic analysis seems to be influenced by ω-3 PUFAs intake, we consider that a real-time lipidomic profilling should be used to monitor the response to ω-3 PUFAs supplementation of T1DM patients and establish intake recommendations in these patients.

## 11 Conclusion

In this review, we highlight the most recent findings on the possible beneficial effects of ω-3 PUFAs regarding the development, progress, and/or prevention of T1DM. There is sufficient evidence to assert that ω-3 PUFAs supplementation could yield significant beneficial effects in the prevention of T1DM and in delaying disease progression. Preclinical reports suggest that the observed effects are related to the anti-inflammatory, antioxidant, antiapoptotic pathways, and the suppression of autoimmunity. Data from clinical and epidemiological studies are heterogeneous, both arguing their health benefits or reporting their absence. However, it must be taken into account the vast differences between study designs and measured outcomes, supplements used, and study cohorts, as most clinical studies comprised small groups with clinical heterogeneity regarding age and the disease stage. Also, when considering the results of these studies, one must take into consideration that the traditional approach to examine the effect of a single nutrient is becoming obsolete. The human diet is complex, and different nutrients could have synergistic or antagonistic effects. For example, vitamin D intake reduces the risk of T1DM in children (Hyppönen et al., 2001), but also co-administration vitamin D and ω-3 PUFAs display benefits for T1DM in the same age group (Cadario et al., 2018; Cadario et al., 2019). In order to truly determine the protective actions of ω-3 PUFAs in T1DM, their effects in the presence and absence of other nutrients, especially those with which they often associate, should be assessed in larger cohort studies, also following the metabolomics (lipidomics, in particular) changes that occur and monitoring the levels pro-/anti-inflammatory molecules and pro-/anti-oxidant balance. These future data will give the opportunity to establish a daily ω-3 PUFAs recommendation for T1DM patients.

## Footnotes

1
http://www.clinicaltrials.gov/show/NCT00333554.
